# Prenatal Heavy Metal Exposure and Adverse Birth Outcomes in Myanmar: A Birth-Cohort Study

**DOI:** 10.3390/ijerph14111339

**Published:** 2017-11-03

**Authors:** Kyi Mar Wai, Ohn Mar, Satoko Kosaka, Mitsutoshi Umemura, Chiho Watanabe

**Affiliations:** 1 Department of Human Ecology, the University of Tokyo, Tokyo, 113-0033, Japan; satoco@humeco.m.u-tokyo.ac.jp; 2 Department of Physiology, University of Medicine 1, Yangon 130404, Myanmar; dr.ohnma@gmail.com; 3 Forestry and Forest Products Research Institute, Forest Research and Management Organization, Tsukuba, Ibaraki 305-8687, Japan; mitsutoshi@affrc.go.jp; 4 National Institute for Environmental Studies, Tsukuba, Ibaraki 305-8506, Japan; chiho.watanabe@nies.go.jp

**Keywords:** heavy metal, cadmium, maternal health, birth outcomes, Myanmar

## Abstract

Arsenic, cadmium and lead are well-known environmental contaminants, and their toxicity at low concentration is the target of scientific concern. In this study, we aimed to identify the potential effects of prenatal heavy metal exposure on the birth outcomes among the Myanmar population. This study is part of a birth-cohort study conducted with 419 pregnant women in the Ayeyarwady Division, Myanmar. Face-to-face interviews were performed using a questionnaire, and maternal spot urine samples were collected at the third trimester. Birth outcomes were evaluated at delivery during the follow up. The median values of adjusted urinary arsenic, cadmium, selenium and lead concentration were 74.2, 0.9, 22.6 and 1.8 μg/g creatinine, respectively. Multivariable logistic regression revealed that prenatal cadmium exposure (adjusted odds ratio (OR) = 1.10; 95% confidence interval (CI): 1.01–1.21; *p* = 0.043), gestational age (adjusted OR = 0.83; 95% CI: 0.72–0.95; *p* = 0.009) and primigravida mothers (adjusted OR = 4.23; 95% CI: 1.31–13.65; *p* = 0.016) were the predictors of low birth weight. The present study identified that Myanmar mothers were highly exposed to cadmium. Prenatal maternal cadmium exposure was associated with an occurrence of low birth weight.

## 1. Introduction

Pregnant women and their fetuses are more vulnerable to adverse effects from the exposure of environmental toxic substances [1,2,3]. Meanwhile, exposure to environmental contaminants during pregnancy may extend negative impacts in early childhood and in later life [4]. Although the placenta may act as a selective transporter that prevents the passage of potentially toxic substances to the developing fetus, some environmental contaminants can freely or partially cross the placental barrier [5]. Particularly, arsenic, cadmium and lead are well-known environmental heavy metals, and they could extend the health risk to the fetus even at a low level through trans-placental circulation [2,6,7,8].

The toxicological effects of heavy metals could alter the physiological changes during pregnancy, the critical phase of fetal cell division and differentiation [4,9]. As an example, prenatal cadmium exposure could impair steroidogenesis that leads to suboptimal fetal growth and development [10]. Lead exposure could interfere with calcium deposition in the bone, resulting in decreased fetal bone growth [11]. Arsenic exposure during pregnancy may also contribute to placental insufficiencies, which could lead to intra-uterine growth retardation through inducing oxidative stress [12].

The associations between prenatal exposure to environmental heavy metals and adverse birth outcomes have been evaluated to varying degrees over the last decades. In many studies, prenatal arsenic, cadmium and lead exposure were inversely associated with anthropological parameters of newborns, such as birth weight, birth length, and head circumference [8,13,14,15,16,17]. Moreover, exposure to these metals also increased the likelihood of preterm delivery [18,19,20]. Exposure to inorganic arsenic during pregnancy was also positively associated with stillbirth and miscarriage [21].

Adverse birth outcomes such as a low birth weight and preterm delivery are closely associated with various lifelong mortality and morbidity risks [22,23]. Generally, low-birth-weight babies are recognizable for higher risks of mortality, morbidity and disability [23]. According to the World Health Organization (WHO), more than 15% of all babies were born with a birth weight under 2500 g, and those from developing countries accounted for more than 95% [23]. The WHO also estimated that the number of low-birth-weight babies was 179 per 1000 live births in Myanmar in 2000 [23]. Preterm delivery is another major determinant of neonatal mortality and morbidity [24]. A study on a total of 4 million early neonatal deaths over 193 countries stated that 28% of neonatal deaths were directly or indirectly due to preterm delivery [25]. A recent systematic analysis showed that the Southeast Asia region accounted for the highest preterm birth rates in 2010, and estimated 13.6% were born preterm [22]. Myanmar is still on the way to progressing in reducing maternal and neonatal mortality. According to the Myanmar Demographic and Health Survey 2015–2016, the estimated infant mortality rate was 40 deaths per 1000 live births and more than 60% of deaths occurred during the first month [26].

In Myanmar, southern and central regions were confirmed to be highly contaminated with arsenic in the ground water [27,28,29,30]. In the southern region of Myanmar, the Ayeyarwady region, of the total 123,964 drinking water samples, 29.18% were found to be above the WHO standard for an arsenic concentration of 10 μg/L and 8.19% of sources exceeded the arsenic concentration of 50 μg/L [28,29]. According to the World Bank Policy report (2005), an estimated 3.4 million of the population were at risk of arsenic contamination in Myanmar [31].

Environmental heavy metal exposure is an emerging public health issue in Myanmar. Previously, arsenic contamination has been confirmed in Myanmar [27,29]. Without a doubt, it is consistently threatening the health of Myanmar people. A previous study in central Myanmar had stated that increased arterial blood pressure and a low brachial index were found among those with nail arsenic level higher than 0.09 µg/g [32]. Additionally, among the populations in the Ayeyarwady region, a negative association was found between 2 h creatinine clearance and a serum arsenic concentration of more than 0.008 µg/L, indicating that chronic arsenic exposure may affect renal glomerular function [33]. Despite the potentials of other heavy metals’ contamination in Myanmar [27,30], an exposure assessment using biological samples has rarely been conducted among the Myanmar population. In addition, there has been no study regarding the extent of prenatal toxicity and the associations between such heavy metal exposure and birth outcomes among pregnant Myanmar women. Therefore, this study aims to examine the prenatal heavy metal exposure by assessing heavy metal concentration in maternal urine, and it also aims to identify the potential effects of prenatal heavy metal exposure on birth outcomes among the Myanmar population.

## 2. Methods

### 2.1. Study Design and Setting

**Figure 1:**
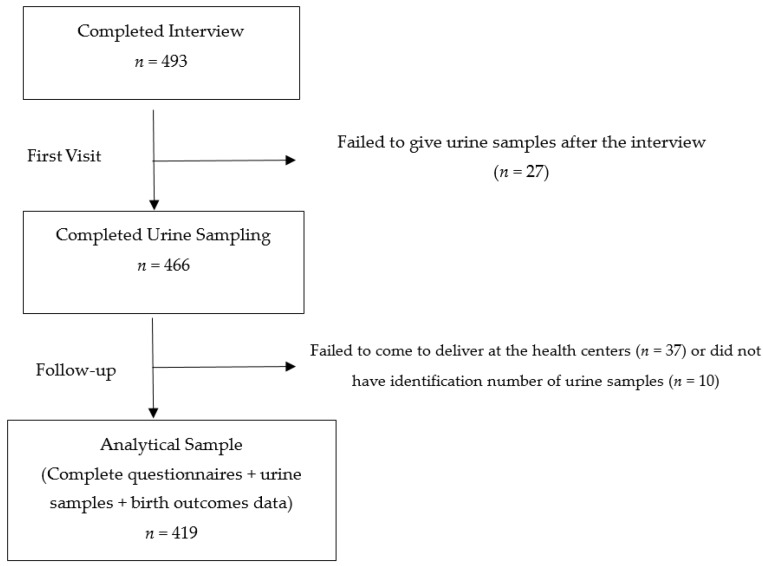
Recruitment and participation in the study.

This study is part of a birth-cohort study conducted in Kyaunggone and Kyonpyaw townships of the Ayeyarwady region in 2016. These townships have been confirmed for high levels of arsenic contamination in ground water [29]. A total of three hospitals from these townships were involved in this study. The included hospitals were township or station-level public hospitals to ensure the accessibility of health services of the general population. The participants were pregnant women aged 18 years or above in their third trimesters, who were residing in the study area for more than six months. A total of 493 participants were recruited during their antenatal visits at the local health centers. In Myanmar, standardized antenatal care is to provide every pregnant woman with at least four antenatal visits with quality care by skilled birth attendants, without any financial burden [34]. According to the recent Demographic and Health Survey 2015–2016, about 78% of women visited the antenatal clinic at least once in the Ayeyarwady region [26]. During the first visit, each participant had undergone a pretested face-to-face questionnaire interview for about 30 to 45 min by the research team. The questionnaire covered background characteristics of participants, pregnancy and birth history. Maternal spot urine samples were also collected at the first visit of the third trimester. All the urine samples were collected in sterile bottles with proper seals and labels. They were firstly stored at −20 °C at the local health centers and then transported to the Department of Human Ecology, the University of Tokyo, Japan under cold chain for further analysis. Only 419 participants were included for the analysis because some participants did not give urine samples after the interview or failed to deliver to the local health centers (Figure 1). During follow up, the information regarding the birth outcomes was extracted from delivery records obtained from the local health centers.

### 2.2. Exposure Assessment

Urinary arsenic, cadmium, lead and selenium concentration were measured using octapole collision/reaction cell inductively coupled-plasma mass spectrometry (ICP-MS; Agilent 7500ce ICP-MS, Agilent Technologies, Santa Clara, CA, USA). Original urine samples were 20-fold diluted with 1% nitric acid (grade for analysis of poisonous metals: 60%; Wako, Osaka, Japan) and 2% 1-butanol (grade for HPLC: 99.5%; Nacalai Tesque, Kyoto, Japan) and filtered through a 0.45 μm pore membrane (Millipore, Billerica, MA, USA) connected to a disposable plastic syringe. Inductively coupled plasma (ICP) multi-element standard solution (XVI CertiPUR, Merck, Darmstadt, Germany) was prepared by a gravimetric method. The detection limit (DL) was calculated as 3 times the standard deviation (SD) of procedural blanks. The average DL values for arsenic, cadmium, lead and selenium were 0.239, 0.025, 0.843 and 0.362 μg/L, respectively. The values under the DL were assumed as a half value of the DL. Analytical quality was assured by the repeated analysis of the samples against National Institute for Environmental Studies (NIES) Certified Reference Material No. 18 Human Urine (NIES, Ibaraki, Japan) and Seronorm Trace Elements Urine (SERO AS, Billingstad, Norway). The certified values of Seronorm Trace Elements Urine for arsenic, cadmium, selenium and lead are 142 μg/L (acceptable range: 130–154 μg/L), 4.6 μg/L (acceptable range: 3.8–5.4 μg/L), 58.6 μg/L (acceptable range: 52.4–64.8 μg/L), and 40.3 μg/L (acceptable range: 35.1–45.6 μg/L), respectively. NIES No.18 Human Urine certified only for limited elements and this study could apply the certified values of arsenic (137 ± 11 μg/L) and selenium (59 ± 5 μg/L). The measured values of the reference materials were within the acceptable ranges. Urinary heavy metal concentration was then adjusted for creatinine. The urinary creatinine concentration was measured by an in vitro colorimetric Jaffe method using a commercial kit (LabAssay Creatinine Kit, Wako, Osaka, Japan).

### 2.3. Measurement of Birth Outcomes and Other Covariates

The pretested questionnaire covered variables including sociodemographic characteristics, anthropological measures, smoking status, pregnancy and obstetric history. Delivery records included information regarding birth, such as the birth weight, the baby’s sex, the mode of delivery, the gestational age and other biological attributes of both the mothers and newborns. Smoking status was categorized as “no exposure at all” or “have or ever been or passively exposed”. In this study, a normal pregnancy outcome is defined as a term delivery without any complication. A low birth weight refers to a birth weight of less than 2500 g at term [23], and preterm delivery refers to live delivery before 37 weeks of completed gestation in accordance with the definition of the International Classification of Diseases by the WHO [24].

### 2.4. Statistical Analysis

Data analysis was performed using Stata 13 (StataCorp LP, College Station, TX, USA). The urinary heavy metal concentration was converted to μg/g creatinine after adjusting for creatinine concentration over the entire analysis. A descriptive analysis was conducted to present means, medians, the interquartile range (IQR), the SD and percentage. A Wilcoxon rank-sum test was applied to compare the exposure levels among different birth outcomes. Multiple logistic regression models were used to identify the associations between prenatal heavy metal exposure and adverse birth outcomes. In the models, the dependent variable was either low birth weight (0 or 1) or preterm delivery (0 or 1). The independent variables included the creatinine-adjusted heavy metal concentration as continuous variables. Potential confounders were also included in the models on the basis of rational associations in previous studies [2,13,19,35]. The statistical significance level was considered as a *p*-value of less than 0.05.

### 2.5. Ethical Considerations

This study was approved by the Research Ethics Committee of the Graduate School of Medicine, the University of Tokyo (No. 11186) and the Department of Medical Research, Myanmar (ERC No. 009316). A material transfer agreement was obtained through the University of Medicine 1, Yangon, Myanmar. All the participants were voluntary, and informed consents were obtained after the explanation of the purposes of the study.

## 3. Results

A total of 493 participants were enrolled during the first visit. Of these, only 419 participants had complete information of delivery and urine samples for analysis in this study (Figure 1). The background characteristics of the participants are presented in Table 1. Of the 419 participants, the mean maternal age was 28 years with a SD value of 6.6. About 74.2% were of Bamar ethnicity, and about 46% had completed primary education. Only 2.1% had smoking history and 49.2% reported passive exposure.

Table 2 presents the information regarding pregnancy and child birth for the participants. The mean gestational week was 38.0 weeks (SD = 2.4). About 67% of the participants received antenatal care more than four times, and their first antenatal visit was at the gestational age of 15.6 weeks (SD = 6.1). Among the newborns, 56.8% were male, while 43.2% were female. Birth weights ranged from 1510 to 6300 g with an average value of 3171.7 g (SD = 493.0). Of the total births, about 19% were premature and 6% were low-birth-weight babies.

Table 3 shows the creatinine-adjusted heavy metal concentrations in maternal urine. The median values of the adjusted maternal urinary concentration of arsenic, cadmium, selenium and lead were 74 μg/g creatinine (IQR: 45–126), 0.86 μg/g creatinine (IQR: 0.50–1.40), 22.5 μg/g creatinine (IQR: 18–30) and 1.75 μg/g creatinine (IQR: 1.0–3.3).

Table 4 presents the comparison of exposure levels among different birth outcomes using the Wilcoxon rank-sum test. The concentration of the adjusted urinary cadmium levels was found to be significantly different between the normal-delivery and low-birth-weight groups (*p* = 0.020). It was also significantly different between normal deliveries and both preterm and low-birth-weight deliveries (*p* = 0.014). There was no significant difference from other heavy metal exposure levels.

Table 5 and Table 6 show the associations between the maternal urinary heavy metal concentration and dichotomous outcomes of a low birth weight and preterm delivery. From bivariate analysis, it was revealed that an increased risk of a low birth weight was associated with a higher maternal urinary cadmium concentration (OR = 1.10; 95% CI: 1.02–1.18; *p* = 0.017). The association was found to be consistent (adjusted OR = 1.10; 95% CI: 1.00–1.21; *p* = 0.043) even after adjusting with maternal age, maternal education, the baby’s sex, smoking status, the gestational age, being primigravida and antenatal visits in multivariate logistic regression. The gestational age (adjusted OR = 0.83; 95% CI: 0.72–0.95; *p* = 0.009) and being primigravida (adjusted OR = 4.23; 95% CI: 1.31–13.64; *p* = 0.016) were also found to be strong predictors of a low birth weight. There was no significant association between the maternal heavy metal concentration and preterm delivery, as shown in Table 6.

## 4. Discussion

This study evaluated the effect of prenatal heavy metal exposure on adverse birth outcomes among the Myanmar population. Overall, the maternal urinary cadmium concentration was statistically different between normal deliveries, low-birth-weight deliveries and deliveries with both preterm and low birth weight. After adjusting for the confounders, this study revealed that a higher maternal urinary cadmium concentration increased the likelihood of a low birth weight but not preterm delivery.

This study firstly examined the maternal urinary heavy metal concentration. The median value of maternal urinary arsenic, cadmium, selenium and lead concentration values were 74.22, 0.86, 22.51, and 1.75 μg/g creatinine, respectively. In comparison with the previous reports, the urinary arsenic concentration among the Myanmar population was much lower than those in the Argentinian population (median = 230 μg/L), the Bangladesh population (mean = 336.7 μg/L), the Nepalese population (mean = 196 μg/g creatinine) and the Indian population (mean = 290 μg/L) [36,37,38,39]. In the case of cadmium, the urinary cadmium concentration in this study (geometric mean (GM) = 0.87 μg/g creatinine) was comparatively higher than in the previous findings on those from Bangladesh (median = 0.63 μg/g creatinine), the United States (mean = 0.46 μg/g creatinine), Nepal (GM = 0.33 μg/g creatinine), China (GM = 0.55 μg/g creatinine) and South Africa (GM = 0.27 μg/g creatinine) [17,19,38,40,41]. The median selenium concentration of this study was lower than 30 μg/g creatinine and was similar to the concentration of the normal reference range [42]. Urinary lead concentration in the present study was also found to be in a similar range to previous reports [14,43].

This study is the first to report a comparatively higher concentration of cadmium among the Myanmar population. Despite smoking being a major source of cadmium exposure, there was no significant association between the smoking status and urinary cadmium concentration in this study. Therefore, it is suggested that the main source of cadmium exposure in Myanmar is diet, either water or food. In the previous study comparing the cadmium content in different food items, the GM for cadmium in rice (50 ng/g) was significantly higher than that of wheat-derived food such as bread (16 ng/g), flour (19.3 ng/g) and noodles (4 ng/g) [44]. Other studies also revealed that cadmium from the soil was absorbed and retained in rice to a great extent [45], and cadmium in rice has been exclusively correlated with cadmium body burden [46]. In Myanmar, rice is the staple food, and other rice-derived foods are also major components of daily meals. Therefore, it is important to trace the potential sources of cadmium contamination while tackling its health concerns in Myanmar.

Several studies have discussed the effect of heavy metal exposure on anthropometric measures of newborns [2,8,13,14,16,47,48]. Particularly, arsenic, cadmium and lead exposure during pregnancy have been significantly associated with a decreased birth weight, even at the low level [8,16,47,48]. Among these, cadmium has been found to have the most distinct effects on several birth outcomes. For example, a study conducted on the Saudi Arabian population stated that the cadmium concentration in umbilical cord blood was inversely correlated with crown-heel length, birth weight, and Apgar 5 min scores and was small for gestational age, whereas the lead or mercury concentration was not associated with these parameters in the same population [8].

A low birth weight is considered as a significant public health concern as it has been highly associated with neonatal mortality and disease risk in adulthood [49]. This study identified the association between the maternal urinary cadmium concentration and an increased likelihood of a low birth weight. The finding is consistent with the previous studies in which prenatal cadmium exposure was associated with the growth of the fetus in utero, leading to a decrease in birth weight among the population exposed to a similar concentration in Bangladesh (median = 0.63 μg/L), Saudi Arabia (mean = 0.99 μg/L) and Japan (GM = 0.77 μg/L) [8,14,48]. The effect was still significant with the lower concentration (GM = 0.25 μg/L) among the South African coastal population, suggesting that even a lower concentration of cadmium exposure may trigger alterations in fetal growth [41]. The underlying mechanisms of cadmium-induced low birth weight have been postulated in many previous studies. Cadmium may interfere with zinc transfer to the fetus, resulting in intrauterine growth retardation [50]. Cadmium also seems to be involved in fetoplacental hormonal alteration, such as in the production of placental progesterone, thyroid stimulation hormone and placental leptin synthesis, which have been linked to impaired fetal growth [10,51,52]. Moreover, experimental studies have supported the evidence that cadmium could impair placental circulation, inhibiting the transport of nutrients from the mother to the fetus [53,54]. In contrast to previous studies, no association was found between the maternal arsenic and lead exposure and a low birth weight in this study [16,47,55]. This could be explained by the comparatively lower concentration of urinary arsenic and lead among our study population, as the exposure dose and timing play a critical role in intrauterine fetal growth [56].

This study also attempted to identify the associations between prenatal heavy metal exposure and preterm delivery. However, in this study, prenatal heavy metal exposure was not significantly associated with preterm delivery. This finding is consistent with the previous report, which stated that the arsenic concentration in drinking water was not significantly associated with an increased risk of preterm delivery in Taiwan [18]. In the case of cadmium, the result was contradictory to the previous findings, which showed that the preterm birth rate was higher with an increased urinary cadmium concentration in China and that the incidence of preterm delivery among those with a higher urinary cadmium concentration (≥2 nmol/mmol creatinine) was higher than among those with a lower cadmium concentration in Japan [19,57]. Regarding lead exposure, the result was in line with a previous study conducted among Swedish and Polish women, which revealed that the lead concentration in the myometrium and placenta was not significantly elevated in preterm delivery compared to term delivery [58], while other studies in China have mentioned that a higher maternal blood lead level (≥10 μg/dL) doubled the risks of preterm delivery [59]. The inconsistencies in the results may be explained by differences in the exposure level among the different populations. The sensitivity of the population may also vary in response to exposure, as toxic effects depend on the variations in the metabolism of heavy metals across different populations [1,60]. Moreover, gestational age was usually estimated to determine preterm delivery on the basis of the date of the last menstrual period and/or ultrasound data [24]. The variability in gestational age by region and assessment protocols may explain the inconsistencies.

This study drew on many important strengths. The study was a birth-cohort prospective design including broad information on potential confounders. The study was conducted at three public general hospitals of the study area to minimize selection bias. The concentration of heavy metals was measured using a sensitive, robust and well-validated method (ICP-MS) and assured with certified reference materials. Multiple important heavy metals were adjusted for rather than the focus being on the exposure of only one heavy metal. This is the first study to report the effect of prenatal heavy metal exposure on birth outcomes among the Myanmar population. Some limitations of this study should also be considered. The present study failed to control the prenatal exposure of other toxic chemicals that may collectively affect birth outcomes. The nutritional intake was not fully considered in this study, although it is an important predictor of a low birth weight [61]. The information on birth outcomes was extracted from the hospitals’ medical records, which may differ according to measurement protocols.

## 5. Conclusions

The present study provides baseline information concerning environmental heavy metal exposure among the Myanmar population. This study also indicates that Myanmar mothers were comparatively highly exposed to cadmium, which should be counted as a public health threat. Prenatal exposure to cadmium was positively associated with the likelihood of a low birth weight in Myanmar. Along with the previous evidence, these results suggest a follow up on whether the effect of early-life exposure on birth weight could increase the health risks in later life. This study clearly highlights the need to consider other health risks of cadmium exposure beyond birth outcomes among the Myanmar population.

## Figures and Tables

**Table 1:** Socioeconomic characteristics of participants (*n* = 419).

Characteristics	*n*	%	Mean	SD
Age (years)	419		27.9	6.6
Religion				
Buddhist	376	89.7		
Christian	41	9.8		
Others	2	0.5		
Ethnicity				
Bamar	311	74.2		
National ethnic groups ^†^	106	25.3		
Others	2	0.5		
Education				
Illiterate	7	1.7		
Able to read and write	66	15.8		
Primary school completed	193	46.1		
Middle school completed	81	19.3		
High school completed	44	10.5		
Graduate and above	28	6.9		
Occupation				
Unemployed or housewives	176	41.9		
Farmers	152	36.3		
Private Sectors	8	1.9		
Government officers	14	3.3		
Own business	32	7.6		
Others	37	8.8		
Hospitals				
Kyaungone	153	36.5		
Kyonpyaw	139	33.2		
Ahtaung	127	30.3		
Monthly household income # (USD)	296		124.2	55.7
Smoking Status				
Not at all	210	50.1		
Have or ever been or passively exposed	209	49.9		

^†^ National ethnic groups include Kachin, Kayar, Kayin, Chin, Mon, Yakhine and Shan. # 1 USD = 1224 MMK as of September 2016.

**Table 2:** Maternal health and delivery record information (*n* = 419).

Characteristics	*n*	%	Mean	SD
Gestational age (weeks)	419		38.0	2.4
Primigravida				
No	181	43.2		
Yes	238	56.8		
Antenatal visits				
Less than four times	136	32.5		
Four or more than four times	283	67.5		
Gestational week of first antenatal visit	419		15.6	6.1
Mode of delivery				
Normal spontaneous delivery	188	44.9		
Assisted delivery ^γ^	7	1.7		
Cesarean delivery	224	53.5		
Baby’s sex				
Male	238	56.8		
Female	181	43.2		
Birth weight (g)	419		3171.7	493.0
Birth outcomes				
Normal alive	329	78.5		
Still-birth	2	0.5		
Preterm ^θ^	80	19.1		
Congenital abnormality	2	0.5		
Low birth weight ^†^	26	6.2		

^θ^ Any delivery before 37 weeks of gestation regardless of birth weight. ^†^ Birth weight <2500 g regardless of gestational age at birth. ^γ^ Assisted delivery includes vacuum or forceps deliveries.

**Table 3:** Heavy metal concentration in maternal urine (*n* = 419).

Adjusted Urinary Heavy Metal Concentration (μg/g Creatinine)	Median	IQR
Arsenic	74	(45–127)
Cadmium	0.86	(0.50–1.40)
Selenium	23	(18–30)
Lead	1.8	(1.0–3.3)

**Table 4:** Comparison of the exposure level with different birth outcomes (*n* = 419).

Adjusted Urinary Heavy Metal Concentration (μg/g Creatinine)	Preterm Delivery ^θ^			Low Birth Weight ^†^			Preterm and Low Birth Weight		
	Yes (*n* = 80)	No (*n* = 339)	*p*-Value	Yes (*n* = 26)	No (*n* = 393)	*p*-Value	Yes (*n* = 18)	No (*n* = 401)	*p*-Value
Arsenic	73.2	74.2	1.000	89.0	73.8	0.500	84.2	73.9	0.490
Cadmium	0.8	0.8	0.743	1.4	0.8	0.020	1.4	0.8	0.014
Selenium	22.4	22.7	0.940	20.6	22.7	0.998	19.3	22.7	0.324
Lead	1.7	1.8	0.729	1.5	1.8	0.117	1.5	1.8	0.236

^θ^ Any delivery before 37 weeks of gestation regardless of birth weight. ^†^ Birth weight < 2500 g regardless of gestational age at birth.

**Table 5:** Associations between urinary heavy metal concentration and low birth weight (*n* = 419).

Characteristics	Crude OR (95% CI)	Adjusted OR (95% CI)
Maternal age (years)	0.99 (0.93–1.05)	1.04 (0.96–1.11)
Maternal education	0.67 (0.45–1.01)	0.72 (0.46–1.13)
Gestational age (weeks)	0.79 (0.69–0.90) ***	0.83 (0.72–0.95) **
Primigravida (ref: non-primigravida)	2.16 (0.89–5.25) *	4.23 (1.31–13.65) *
Antenatal visit ≥4 times (ref: <4 times)	0.54 (0.24–1.20)	0.55 (0.22–1.36)
Cesarean section or assisted delivery (ref: normal vaginal delivery)	0.68 (0.31–1.51)	0.67 (0.28–1.63)
Baby’s sex (ref: male)	1.58 (0.71–3.50)	1.60 (0.67–3.85)
Have or ever been or passively exposed to smoking (ref: no exposure)	1.01 (0.42–2.22)	0.76 (0.32–1.82)
Arsenic concentration (μg/g creatinine)	1.00 (0.99–1.00)	0.99 (0.99–1.00)
Cadmium concentration (μg/g creatinine)	1.01 (1.02–1.19) *	1.10 (1.01–1.21) *
Selenium concentration (μg/g creatinine)	1.02 (0.99–1.04)	1.02 (0.99–1.06)
Lead concentration (μg/g creatinine)	0.85 (0.67–1.08)	0.76 (0.57–1.03)

* *p* < 0.05; ** *p* < 0.01; *** *p* < 0.001.

**Table 6:** Associations between urinary heavy metal concentration and preterm delivery (*n* = 419).

Characteristics	Crude OR (95% CI)	Adjusted OR (95% CI)
Maternal age (years)	0.99 (0.96–1.04)	1.02 (0.97–1.07)
Maternal education	0.88 (0.70–1.10)	0.92 (0.72–1.19)
Birth weight (grams)	1.00 (0.997–0.999) ***	0.52 (0.44–0.62) ***
Primigravida (ref: non-primigravida)	1.34 (0.81–2.21)	1.30 (0.66–2.57)
Antenatal visit ≥4 times (ref: <4 times)	1.24 (0.73–2.11)	1.59 (0.88–2.89)
Cesarean section or assisted delivery (ref: normal vaginal delivery)	0.41 (0.25–0.68) **	0.40 (0.23–0.70) *
Baby’s sex (ref: male)	1.24 (0.76–2.02)	1.17 (0.68–2.00)
Have or ever been or passively exposed to smoking (ref: no exposure)	1.56 (0.95–2.55)	1.56 (0.91–2.69)
Arsenic concentration (μg/g creatinine)	1.00 (0.99–1.00)	1.00 (0.99–1.00)
Cadmium concentration (μg/g creatinine)	1.06 (0.98–1.14)	1.05 (0.97–1.13)
Selenium concentration (μg/g creatinine)	0.99 (0.98–1.02)	0.99 (0.96–1.01)
Lead concentration (μg/g creatinine)	0.96 (0.87–1.05)	0.98 (0.89–1.07)

* *p* < 0.05; ** *p* < 0.01; *** *p* < 0.001.
